# Colorimetric RT-LAMP for SARS-CoV-2 detection from nasopharyngeal swabs or crude saliva: a multicountry diagnostic accuracy study in Africa

**DOI:** 10.1016/S2214-109X(25)00150-0

**Published:** 2025-06-25

**Authors:** Urša Šušnjar, Molalegne Bitew, Samuele Ayele, Tina Uršič, Miroslav Petrovec, Tea Carletti, Erica Bussani, Laura De Conti, Feleke Mekonnen, Marycelin Mandu Baba, Eric Lelo Agola, Maria Madalena Chimpolo, Joaquim Carlos Vicente Van-Dúnem, Zékiba Tarnagda, Solange Kakou-Ngazoa, Djibril Wade, Kenneth Kudzai Maeka, Mubarak Mustafa, Ralitsa Raycheva, Jean Kaseya, Nicaise Ndembi, Joseph Fokam, Alessandro Marcello, EXPANDIA Working Group, Nasser Caiombo Calumbuana, Elka Kay, Valdemar José Mateus, Joana Paulo Paixão, Faustino Manuel Ramos, Joaquim Carlos Vicente Van-Dunem, Maria Madalena Chimpolo, Assana Cisse, W. O. Benjamin Kabore, Moussa Lingani, Evelise Augusto Machado, Jean Bienvenue Ouoba, Grissoum Tarnagda, Zékiba Tarnagda, Collins Ambe Chenwi, Aude Christelle Ka'e, Aurelie Minelle Ngueko Kengni, Aissatou Abba, Grace Beloumou, Takou Desire, Sandrine Djupsa, Naomi-Karell Etame, Nadine Nguendjoung Fainguem, Ezechiel Ngoufack Jagni Semengue, Nnomo Zam Marie Kryste, Larissa Gaelle Moko, Evariste Molimbu, Alexis Ndjolo, Alex Durand Nka, Michel Carlos Tchouaket Tommo, Joseph Fokam, Adugna Abera, Getachew Abichu Duressa, Kominist Asmamaw Anley, Gadissa Bedada Hundie, Hailu Dadi, Feleke Mekonnen, Yared Merid, Andaragachew Mulu, Kassahun Tesfaye, Keyru Tuki, Gizachew Yismaw, Molalegne Bitew, Jean Luc Frejus Aboh Kamenan, Serges Camara, M. Anicet Ebou, Sylvie Mireille Kouamé-Sina, Dosso Mireille, Aboubacar Sylla, Albert Konan Yavo, Solange Kakou-Ngazoa, Wallace Dimbuson Bulimo, Janet Masitsa Majanja, Samwel Lifumo Symekher, Eric Agola Lelo, Ibrahim Kidda, Tijani Abdulkadir, Ahmed Ahidjo, Musa Joseph Bamaiyi, Bamidele Soji Oderinde, Marycelin Mandu Babaa, Mame Matar Diop, Ndeye Dieynaba Diouf, Nafissatou Leye, Djibril Wade, Asrar M Abdelsalam, Rasheeda H A Ahmed, Hanan Mohamed, Musab Elnegoumi, Nuha Yousif, Shahinaz Bedri, Mohamed Bashir, Fathi Mansour, Lubna Tagelsir karamalla Tagelsir karamalla, Mubarak Mustafa, David Amini, Ezekiel Dhitima, Hlanai Gumbo, Agnes Juru-Chibango, Tapfumanei Mashe, Charles Nyagupe, Raiva Simbi, Lucia Sisya, Kenneth Kudzai Maeka, Tea Carletti, Laura De Conti, Erica Bussani, Mariana Ulinici, Urša Šušnjar, Alessandro Marcello, Tina Uršič, Miroslav Petrovec

**Affiliations:** a International Centre for Genetic Engineering and Biotechnology, Trieste, Italy; b Bio and Emerging Technology Institute, Addis Ababa, Ethiopia; c Armauer Hansen Research Institute, Addis Ababa, Ethiopia; d Institute of Microbiology and Immunology, Faculty of Medicine, University of Ljubljana, Ljubljana, Slovenia; e College of Medicine and Health Sciences, Bahir Dar University, Bahir Dar, Ethiopia; f University of Maiduguri Teaching Hospital, Maiduguri, Nigeria; g Kenya Medical Research Institute, Nairobi, Kenya; h Faculdade de Medicina da Universidade Agostinho Neto, Luanda, Angola; i Institut de Recherche en Sciences de la Santé, Ouagadougou, Burkina Faso; j Institut Pasteur de Côte d’Ivoire (IPCI), Abidjan, Côte d’Ivoire; k Institute for Health Research—Epidemiological Surveillance and Training, Dakar, Senegal; l National Microbiology Reference Laboratory, Harare, Zimbabwe; m Tropical Medical Research Institute, National Centre for Research, Khartoum, Sudan; n Medical University Plovdiv, Plovdiv, Bulgaria; o Africa Centres for Disease Control and Prevention, Addis Ababa, Ethiopia; p Institute of Human Virology, Baltimore, MD, USA; q Chantal Biya International Reference Centre for Research on HIV/AIDS Prevention and Management, Yaounde, Cameroon; r Department of Medical Laboratory Science, Faculty of Health Sciences, University of Buea, Buea, Cameroon

## Abstract

**Background:**

Reverse-transcription loop-mediated isothermal amplification (RT-LAMP) with colorimetric readout is a rapid, robust, and cost-effective one-step amplification assay that we previously trialled for the identification of SARS-CoV-2 in nasopharyngeal swabs in four countries. Here, we expanded our assessment of RT-LAMP for SARS-CoV-2 detection to several other African countries and evaluated its operational performance with crude saliva as a pragmatic approach for outbreak surveillance and response in resource-limited settings.

**Methods:**

We conducted a multicountry diagnostic accuracy study of RT-LAMP for the detection of SARS-CoV-2 in different types of clinical samples. A preliminary study was conducted in Slovenia and Italy to establish the analytical performance (limit of detection) of RT-LAMP and optimise this assay before its deployment in Africa. Subsequently, we tested RT-LAMP with RNA extracted from nasopharyngeal swabs in seven countries in Africa (Angola, Burkina Faso, Côte d’Ivoire, Ethiopia, Senegal, Sudan, and Zimbabwe), and, in parallel, with crude saliva samples (ie, without RNA extraction) in an additional four countries (Cameroon, Ethiopia, Kenya, and Nigeria; paired nasopharyngeal swabs were collected at the same time). In both contexts, quantitative RT-PCR (RT-qPCR) with RNA extracted from nasopharyngeal swabs was used as the gold-standard benchmarking assay to evaluate performance. For RT-qPCR testing, each laboratory followed their own standard diagnostic procedure, whereas a standardised protocol was used for RT-LAMP. Saliva test standardisation was ensured through centralised reagent distribution. We calculated diagnostic parameters (sensitivity, specificity, and accuracy) using a 2 × 2 contingency table.

**Findings:**

The preliminary study reported 87% sensitivity and 98% specificity for RT-LAMP. Between Sept 1, 2021, and June 30, 2022, we collected 2774 nasopharyngeal swabs and 577 crude saliva samples. For RNA extracted from nasopharyngeal swabs, the sensitivity and specificity of RT-LAMP for detection of SARS-CoV-2 (relative to the standard of diagnostics—ie, the RT-qPCR assay used in each participating laboratory) were 89% (95% CI 87–90) and 95% (93–96), respectively. Similarly, RT-LAMP tested on saliva without RNA extraction showed 80% (75–84) sensitivity and 99% (96–100) specificity (relative to the results obtained with the standard of diagnostics for RNA extracted from paired nasopharyngeal samples).

**Interpretation:**

Colorimetric RT-LAMP is a reliable assay for SARS-CoV-2 detection in both extracted RNA and crude saliva samples. The demonstrably acceptable performance on crude saliva samples (without RNA extraction) underscores the scalability of this method for efficient outbreak surveillance in resource-limited settings.

**Funding:**

Gates Foundation.

## Introduction

The COVID-19 pandemic caused by SARS-CoV-2 remains a global public health concern.^1^ Timely and reliable on-site virus identification is key to the effective management of outbreaks, highlighting the need for tests that are cost-effective and deployable in resource-limited settings.^2^, ^3^ Real-time quantitative RT-PCR (RT-qPCR) requires specialised instruments, expertise, and time and might not be used as a point-of-care diagnostic method in such settings.^4^ Portable quantitative PCR machines based on pre-loaded chips, which would partially solve the instrumentation problem, have been developed in recent years. Although they are sensitive and specific, the costs of the machines, as well as their electricity dependency and requirement for personnel training for result interpretation, remain non-negligible hurdles.^5^, ^6^

Reverse transcription loop-mediated isothermal amplification (RT-LAMP), which has a colorimetric readout, has the potential to contribute to rapid outbreak surveillance and control in resource-limited settings. Several studies have shown promising performance for RT-LAMP in detecting SARS-CoV-2, with accuracy ranging from 71% to 100%, compared with RT-qPCR gold standard.^7^, ^8^, ^9^, ^10^ We previously explored the application of RT-LAMP in different contexts within Africa to increase awareness of the method in the public health sector and provide a viable alternative to RT-qPCR.^11^ Although the latter remains the gold standard for SARS-CoV-2 diagnostics, RT-LAMP-based assays have been shown to be superior to rapid diagnostic tests in terms of sensitivity.^4^, ^12^, ^13^, ^14^

**Research in context**

**Evidence before this study**

We searched PubMed for original research articles published from database inception to Dec 12, 2022, investigating the diagnostic performance of various reverse transcription loop-mediated isothermal amplification (RT-LAMP) assays for detecting SARS-CoV-2, without language restrictions. Using the search terms (“RT-LAMP” OR “loop-mediated isothermal amplification”) AND (“diagnostic performance” OR “diagnostic accuracy”) AND (“SARS-CoV-2” OR “COVID-19”), we found 21 relevant studies. We identified additional publications on the topic by expanding our search to Google Scholar. Most studies focused on developing or evaluating RT-LAMP assays for SARS-CoV-2 detection. Some aimed to optimise primer combinations for variant detection, whereas others explored innovative detection techniques, such as colorimetric methods, fluorometric probe-based methods, or CRISPR-based methods, and extraction-free or automated protocols. Reviews and meta-analyses published at the time indicated that the diagnostic sensitivity of individual RT-LAMP assays was generally high (usually >85% compared with reference methods). With only a few exceptions, most studies were conducted in well-equipped laboratories in developed countries.

**Added value of this study**

The key strength of this study lies in its comprehensive multicentre and multicountry clinical evaluation of a molecular diagnostic tool—a less commonly used alternative to the gold-standard quantitative RT-PCR—specifically in and for Africa. We assessed RT-LAMP with both nasopharyngeal swabs and direct saliva samples and established a robust framework for in-field testing. Several recent reports have emphasised the importance of strengthening clinical trial capacity in Africa, highlighting it as a crucial step in enhancing pandemic preparedness across the continent. Multicentric clinical trials are essential for the thorough evaluation of new diagnostic methods, not only because they involve a larger number of participants but also because they account for variations in laboratory infrastructure, reliability of electricity supplies, sample collection procedures, and the training levels of laboratory personnel. These trials capture the real-world challenges faced by different laboratories, particularly community laboratories in low-income and middle-income countries.

**Implications of all the available evidence**

The diagnostic performance and key attributes of the colorimetric RT-LAMP assay make it well suited for point-of-care use in low-resource settings, potentially with applications beyond SARS-CoV-2 detection. In addition to clinically evaluating this RT-LAMP assay, we have been collaborating with regulatory bodies to provide sufficient evidence for the recognition of this technique as a valid diagnostic test in partner countries. Notably, the assay has already been approved for clinical diagnosis in two partner countries. The need for such diagnostic tools, along with streamlined strategies for the rapid assessment of emerging infectious diseases, is more urgent than ever, as underscored by the ongoing emergencies.

Of the various methods of sample collection for SARS-CoV-2 detection, collection of saliva is less invasive than collection of nasopharyngeal swabs; it is also compatible with self-sampling and requires no specialised consumables.^15^, ^16^, ^17^, ^18^ Thus, saliva testing could be a suitable alternative first-line screening test in several settings, including resource-limited settings, preserving nasopharyngeal swabs for patients with specific clinical indications.^19^, ^20^, ^21^ Many trials have shown that saliva is compatible with RT-LAMP even without RNA purification.^15^, ^22^, ^23^, ^24^, ^25^, ^26^, ^27^ The minimal upstream processing needed for crude saliva greatly reduces the overall cost and time taken for testing, ensuring successful deployment of RT-LAMP in resource-limited settings for pandemic control.^15^

To assess the diagnostic performance of RT-LAMP for detection of SARS-CoV-2 in both nasopharyngeal swabs and crude saliva samples, we conducted an extended field assessment in several countries in Africa, including peripheral health-care locations.

## Methods

### Study overview

**Figure 1:**
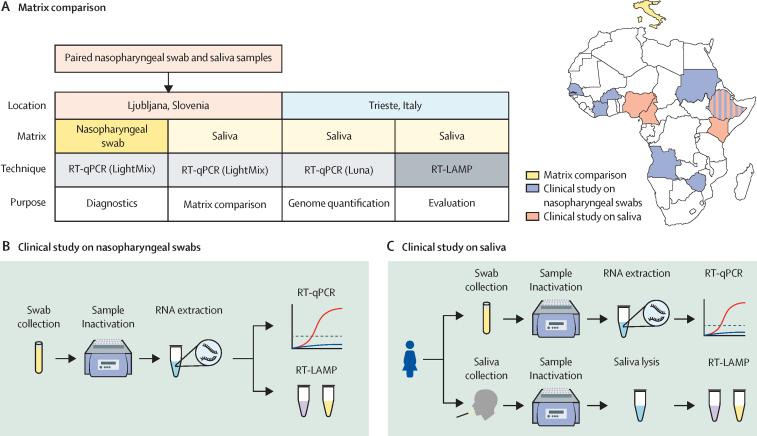
Study overview (A) Study scheme (left) and locations of participating countries conducting the two clinical trials (right). (B) Procedure for the clinical study on nasopharyngeal swabs; figure created with Biorender.com. (C) Procedure for the clinical study on saliva; figure created with Biorender.com. RT-LAMP=reverse transcription loop-mediated isothermal amplification. RT-qPCR=quantitative RT-PCR.

The RT-LAMP protocol using extracted RNA from nasopharyngeal swabs was established previously in a small-scale, multicentric study conducted in four reference laboratories in Africa,^11^ whereas the protocol for SARS-CoV-2 detection in crude saliva had yet to be defined. Thus, we conducted a preliminary study to assess the performance of RT-LAMP in crude saliva samples without previous RNA purification. This study was conducted in Slovenia and Italy to establish the analytical performance (limit of detection) of RT-LAMP and to optimise this assay before its deployment in Africa. Subsequently, two multicountry diagnostic trials were conducted in Africa to assess the performance of RT-LAMP in detecting SARS-CoV-2. RNA samples extracted from nasopharyngeal swabs were used for the assessment in laboratories in seven African countries (hereafter referred to as the clinical study on nasopharyngeal swabs). Crude lysed saliva samples without RNA extraction (hereafter referred to as the clinical study on saliva) were assessed in four other African countries (figure 1).

### Sample size estimation

At the time of trial planning, few SARS-CoV-2 prevalence data were available for the countries in the study. We conservatively estimated a prevalence of 5%, aligning with data from the Africa Centres for Disease Control and Prevention (Africa CDC; updated on Jan 1, 2023), Slovenian sample collection data, and published RT-qPCR reports (appendix p 1). Based on the sensitivity and specificity of the RT-LAMP assay^11^ and a 5% margin of error at a 95% confidence level, each study required 915 samples.^28^ We aimed for 900 samples per study but exceeded this number when Ethiopian community laboratories joined, with testing of nasopharyngeal swabs.

### Sample collection for the preliminary matrix comparison

Participants were recruited from Slovenia's largest COVID-19 swab centre (Community Health Centre, Ljubljana). Participants self-collected ≥1 mL of posterior saliva under medical supervision and completed a clinical symptom questionnaire (appendix p 2);^21^ nasopharyngeal swabs were collected at the same time by community health centre personnel. Nasopharyngeal swabs were collected with the CITOSWAB virus collection and transport kit (Nal von Minden, Moers, Germany) and saliva samples were collected with saliva collectors (Biocomma, Shenzhen, China). Samples were analysed immediately after collection, in a different laboratory at the same location. Ethics approval was provided by the Committee of the Republic of Slovenia for medical ethics, at the Ministry of Health (Slovenia). Written informed patient consent was obtained from all individuals providing samples before collection.

### RNA extraction and RT-qPCR for clinical diagnosis

For direct comparison, nasopharyngeal swabs and saliva samples were processed in the same manner, with no reagents added to the saliva samples. RNA was isolated from 200 μL of saliva or from the nasopharyngeal swab with Nextractor NX-48S (Genolution, Seoul, South Korea), with 10 μL of equine arteritis virus added as an internal control. The CE IVD LightMix kit SARS-CoV-2 E+N UBC (TIB Molbiol, Berlin, Germany) was used for RT-qPCR, according to the manufacturer's instructions. SARS-CoV-2 RNA and human DNA were quantified using gBlocks Gene Fragments (Integrated DNA Technologies, Coralville, IA, USA) and an in-house standard.

### RNA extraction and genome quantification

After clinical testing was done in Slovenia, crude saliva samples were frozen (–80°C) and shipped to the International Centre for Genetic Engineering and Biotechnology ([ICGEB], Trieste, Italy). The samples were thawed and heat inactivated (30 min at 65°C), and viral RNA was extracted from 140 μL of saliva with the QIAamp Viral RNA Mini Kit (Qiagen, Hilden, Germany) and eluted in 60 μL of water. SARS-CoV-2 genome copies were quantified via a standard curve generated with synthetic viral RNA^29^ and an international SARS-CoV-2 standard. The Luna Universal Probe One-Step RT-qPCR kit (New England Biolabs, Ipswich, MA, USA) was used for RT-qPCR targeting the SARS-CoV-2 *N* locus (DrostenN primers; forward: 5ʹ-CACATTGGCACCCGCAATC-3ʹ [0·6 μmol/L]; reverse: 5ʹ-GAGGAACGAGAAGAGGCTTG-3ʹ [0·8 μmol/L]; DrostenN probe 5ʹ-FAM-ACTTCCTCAAGGAACAACATTGCCA-BBQ-3ʹ [0·2 μmol/L]), with the following amplification protocol: 55°C (10 min), 95°C (3 min), followed by 45 cycles of 95°C (15 s) and 58°C (30 s).

### Limit of detection

The limit of detection for RT-LAMP was established using serial dilutions of the WHO SARS-CoV-2 standard (National Institute for Biological Standards and Control) in pooled negative saliva or Copan medium. Theoretical limit of detection was defined as the RNA concentration detected in ≥95% of cases (19 of 20); clinical limit of detection was the concentration at which RT-LAMP detected SARS-CoV-2 with 100% sensitivity in crude saliva (clinical samples).

### Saliva lysis and RT-LAMP

Heat-inactivated saliva (15 μL) was mixed with 2X saliva lysis buffer,^30^ heated at 95°C for 5 min, and used for RT-LAMP with the SARS-CoV-2 Rapid Colorimetric LAMP Assay Kit (New England Biolabs, Ipswich, MA, USA), targeting the *E* and *N* loci. The colour change (pink to yellow) was assessed after 30 min at 65°C (appendix p 8). Results of RT-LAMP were deemed inconclusive for samples that were either positive or negative by RT-qPCR but for which the colour change with colorimetric RT-LAMP was unclear to the operator.

### Clinical study on nasopharyngeal swabs

This multicountry, cross-sectional study in Africa evaluated nasopharyngeal swab samples collected from individuals in seven countries (Angola, Burkina Faso, Côte d’Ivoire, Ethiopia, Senegal, Sudan, and Zimbabwe). In Ethiopia, samples were analysed in seven decentralised community laboratories as well as in the central reference laboratory (figure 1B). Details of the sites of collection and participating laboratories are in the appendix (p 3). Samples were collected prospectively when possible or retrieved from storage and retested using RT-qPCR. RT-LAMP was done on extracted RNA alongside RT-qPCR, to minimise bias from freeze–thaw cycles. Written informed consent was obtained from all individuals providing samples before collection; details of ethical approvals are in the appendix (p 3). For RT-qPCR testing, each laboratory followed their own standard diagnostic procedure (reference test), whereas RT-LAMP was conducted following the proposed protocol provided by the ICGEB.

### Clinical study on saliva

In this pilot study, conducted in Cameroon, Ethiopia, Kenya, and Nigeria, paired nasopharyngeal swabs and saliva samples were collected for analysis (figure 1C). Ethical approval and informed consent were obtained before sample collection (appendix p 4). Nasopharyngeal swabs underwent RNA extraction and RT-qPCR testing according to the standard RT-qPCR procedure in place in each laboratory (appendix p 4). On the basis of the RT-qPCR results for the nasopharyngeal swabs, corresponding paired saliva samples (100 positive samples and 50 negative samples; 30% of positive samples had cycle thresholds [Ct] >30) were to be chosen for RT-LAMP testing. Before testing with RT-LAMP, samples were randomised and de-identified. A standardised, simplified RT-LAMP protocol provided by the ICGEB for the direct processing of crude saliva samples was used for the 150 eligible saliva samples. Saliva test standardisation was ensured through centralised reagent distribution. Numbers of samples collected in each country varied due to declining numbers of COVID-19 cases.

### Statistical analysis

Data were collected with a standardised case report form and processed in Microsoft Excel and Stata (v17). Inconclusive samples were excluded. We calculated diagnostic parameters (sensitivity, specificity, and accuracy) using a 2 × 2 contingency table with the epiR package in R (v 4.3.1). Cohen's Kappa coefficient was used to assess agreement between RT-LAMP and RT-qPCR. Ct values obtained by RT-qPCR with nasopharyngeal swabs or saliva were compared with the paired Student's *t* test, and Pearson correlation was used to measure SARS-CoV-2 concentrations across matrices. Viral loads in different matrices were assessed by comparing Ct values obtained from RT-qPCR (raw values or values normalised for an internal control Ct [*UBC*]) on RNA extracted from nasopharyngeal swabs or saliva; a 5% significance level was applied.

### Role of the funding source

The funder of the study had no role in study design, data collection, data analysis, data interpretation, or writing of the report.

## Results

338 individuals were enrolled in Ljubljana, Slovenia, during the delta (Sept 9–Oct 30, 2021; n=296) and omicron (Jan 1–19, 2022; n=42) variant waves of COVID-19 to obtain paired nasopharyngeal swabs and saliva samples for the matrix comparison study (figure 1A). On the basis of RT-qPCR results obtained with the nasopharyngeal swabs (assessed in the reference laboratory), 200 (59%) of these individuals were diagnosed as positive for SARS-CoV-2 and 138 (41%) were diagnosed as negative. Among the positive samples, 60 (30%) had low viral loads (Ct >25); appendix p 8).

**Figure 2:**
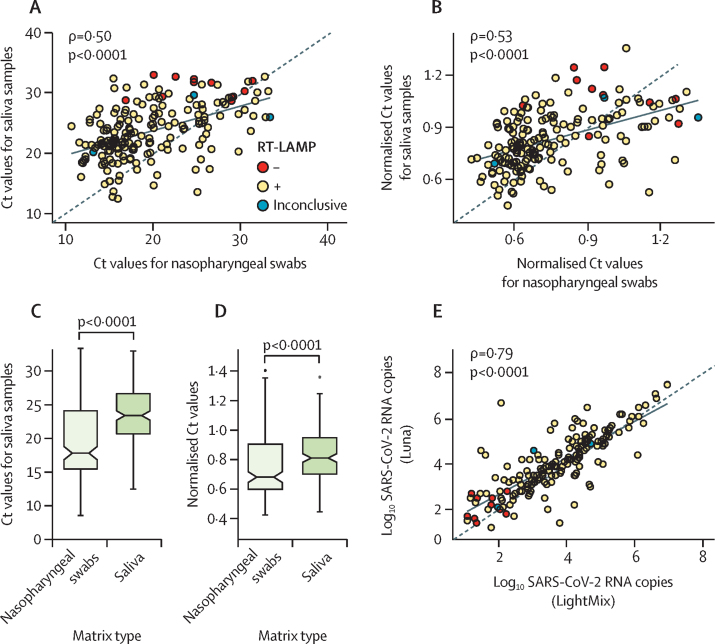
Viral loads in paired nasopharyngeal swab and saliva samples (A) Scatter plot shows low correlation between Ct values for paired nasopharyngeal swab and saliva samples from individuals diagnosed positive for SARS-CoV-2 as assessed by RT-qPCR (n=186). (B) Ct values from the plot in panel A were normalised for an internal control (*UBC*) in each sample (n=177; nine samples were excluded as no signal was obtained for *UBC*). (C) Box plot of Ct values from panel A. Boxes indicate limits of the first and third quartile; lines show the median; whiskers represent 1·5-times the IQR. (D) Ct values from panel C were normalised for an internal control (*UBC*) in each sample (n=177). Boxes indicate limits of the first and third quartile; lines show the median; whiskers represent 1·5-times the IQR. (E) Correlation of viral loads in positive saliva samples as assessed by RT-qPCR using two different assays in two laboratories at distinct timepoints (n=188; five samples were excluded from quantification as saliva volume was insufficient for additional analysis). Dashed lines in A, B, and E represent y=x. Ct=cycle threshold.RT-LAMP=reverse transcription loop-associated isothermal amplification.

As different sample types have previously been reported to contain variable concentrations of the virus,^31^ viral loads were first assessed in the two matrices by RT-qPCR, with RNA extracted from nasopharyngeal swabs or paired saliva samples in the same diagnostic facility. Nasopharyngeal swabs yielded more positive samples (200 [59%] of 338) than did the paired saliva samples (193 [57%]). Nonetheless, there was poor correlation between Ct values obtained for nasopharyngeal swabs and saliva samples from SARS-CoV-2-positive individuals (figure 2A), even when the viral concentration (ie, Ct values for *N* and *E* genes) was normalised to *UBC* expression to control for the amount of human material in each sample (figure 2B). In our sample set, viral loads were higher for nasopharyngeal swabs than for the paired saliva samples (figure 2C; difference in means 3·9 Ct, p<0·0001), a difference that has been reported previously.^19^ The same trend was observed when Ct values for SARS-CoV-2 were normalised to *UBC* expression (difference in means 0·07 Ct, p<0·0001; figure 2D). Although viral load itself depended on the sample type, viral SARS-CoV-2 RNA showed good stability in saliva, as RT-qPCR conducted on RNA samples extracted from patient saliva in two different facilities on different days (samples underwent a freeze–thaw cycle) produced consistent results (Pearson's *r*=0·79, p<0·0001; figure 2E).

**Figure 3:**
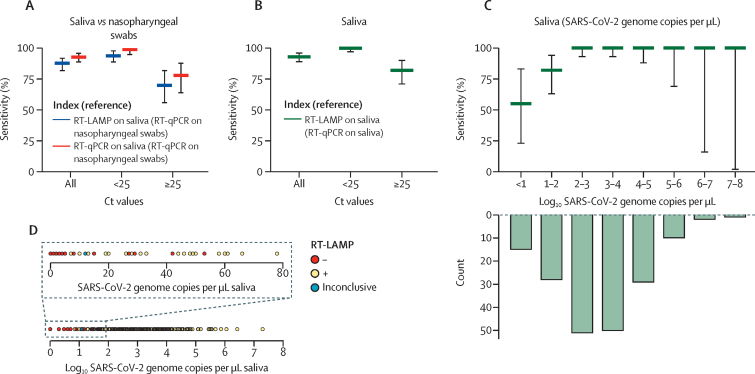
Sensitivity of RT-LAMP for detection of SARS-CoV-2 in crude saliva samples (A) Sensitivity of RT-LAMP with crude saliva samples and RT-qPCR (LightMix) with RNA purified from saliva samples relative to the reference diagnostic method, stratified by RT-qPCR Ct values; n=144 for Ct <25; n=53 for Ct ≥25 (three positive samples [as assessed by RT-qPCR] were excluded due to an inconclusive colour change in RT-LAMP). (B) Sensitivity of RT-LAMP with crude saliva samples relative to RT-qPCR (LightMix) on RNA extracted from saliva, stratified by RT-qPCR Ct values. (C) Sensitivity of RT-LAMP as in panel A stratified by the number of genome copies per μL of saliva (upper plot) quantified with RT-qPCR (Luna); number of samples in each concentration range (count; lower plot). (D) Limit of detection of RT-LAMP for crude saliva samples lysed in saliva lysis buffer (n=193 SARS-CoV-2-positive individuals, as diagnosed by RT-qPCR on RNA extracted from saliva). Sensitivity values (thick lines) in A–C are shown with 95% CIs (whiskers). RT-LAMP=reverse transcription loop-mediated isothermal amplification. Ct=cycle threshold. RT-qPCR=quantitative RT-PCR.

The sensitivity of RT-LAMP for detection of SARS-CoV-2 in crude saliva samples, assessed against the gold-standard diagnostic procedure (ie, RT-qPCR run on RNA extracted from nasopharyngeal swabs), was 88% (95% CI 82–92; table 1, figure 3A). Four samples were excluded from the evaluation of assay sensitivity because of inconclusive RT-LAMP colour changes. The sensitivity of RT-LAMP was slightly lower than the sensitivity of RT-qPCR with crude saliva samples (table 1).

**Table 1:** Diagnostic parameters of RT-LAMP or RT-qPCR on crude saliva samples relative to the reference method

	**Reference or benchmarking assay**	**Number of samples**	**Sensitivity (95% CI)**	**Specificity (95% CI)**
Saliva (RT-LAMP)	Nasopharyngeal swab (RT-qPCR)	334	88% (82–92)	95% (90–98)
Saliva (RT-qPCR)	Nasopharyngeal swab (RT-qPCR)	338	93% (89–96)	95% (90–98)
Saliva (RT-LAMP)	Saliva (RT-qPCR)	334	93% (89–96)	98% (94–100)

RT-LAMP=reverse transcription loop-associated isothermal amplification. RT-qPCR=quantitative RT-PCR.

As expected, the sensitivity of RT-LAMP was higher for samples with high viral loads (94%, Ct <25) than for those with a lower viral concentration (70%, Ct ≥25). Additionally, when the results of RT-LAMP on crude saliva samples were benchmarked against RT-qPCR with RNA extracted from saliva, relative sensitivity was higher for high viral loads (99% [95% CI 97–100]) than for low viral loads (78% [71–80]; figure 3B). The threshold between high and low viral loads was arbitrarily set at a Ct value of 25 because the diagnostic kit amplified two SARS-CoV-2 genes within the same channel, thus generating lower Ct values than diagnostic kits with a single target in individual channels.

The sensitivity of RT-LAMP for detection of SARS-CoV-2 in crude saliva samples increased to 93% (95% CI 89–96) when the reference method (RT-qPCR) used RNA samples extracted from saliva rather than from nasopharyngeal swabs (ie, a direct comparison of of the performances of the two diagnostic tools for detecting SARS-CoV-2 in the same matrix; figure 3B, table 1). Despite a relatively low RT-LAMP sensitivity (55% [23–83]) for saliva with <10 genome copies per μL, the assay attained 100% sensitivity for samples with a viral concentration of >100 genome copies per μL (figure 3C).

The clinical limit of detection, defined as the concentration of viral RNA at which the RT-LAMP assay would reliably and reproducibly detect SARS-CoV-2 in clinical saliva samples, was 58 SARS-CoV-2 genome copies per μL of saliva (figure 3D, table 2). This limit was consistent with the theoretical limits of detection for saliva or nasopharyngeal swabs as assessed with a series of dilutions of the WHO international standard for SARS-CoV-2 in either pooled SARS-CoV-2-negative saliva or Copan medium. This preliminary work enabled the streamlining of the protocol for paired collection and processing of clinical samples, facilitating the subsequent clinical studies.

**Table 2:** Limits of detection of the RT-LAMP assay

	**Protocol**	**Standard**	**Copies of SARS-CoV-2 genome per reaction**	**Ct**
**Theoretical limit of detection**				
Nasopharyngeal swab	RNA extraction	International standard	35	35·96
Saliva	Lysis in saliva lysis buffer	International standard	38	35·84
**Clinical limit of detection**				
Saliva	Lysis in saliva lysis buffer	NA	58	35·23

Ct=cycle threshold. NA=not applicable**.** RT-LAMP=reverse transcription loop-mediated isothermal amplification.

In the clinical study on nasopharyngeal swabs, 2774 swabs were collected, of which 1522 (54·9%) were positive for SARS-CoV-2 by RT-qPCR. Inconclusive RT-LAMP results were recorded in 80 (2·9%) samples (appendix p 5). The overall sensitivity of colorimetric RT-LAMP relative to the sensitivity of RT-qPCR was 89% (87–90), specificity was 95% (93–96), and accuracy was 92% (90–93; appendix p 5 figure 4A).

**Figure 4:**
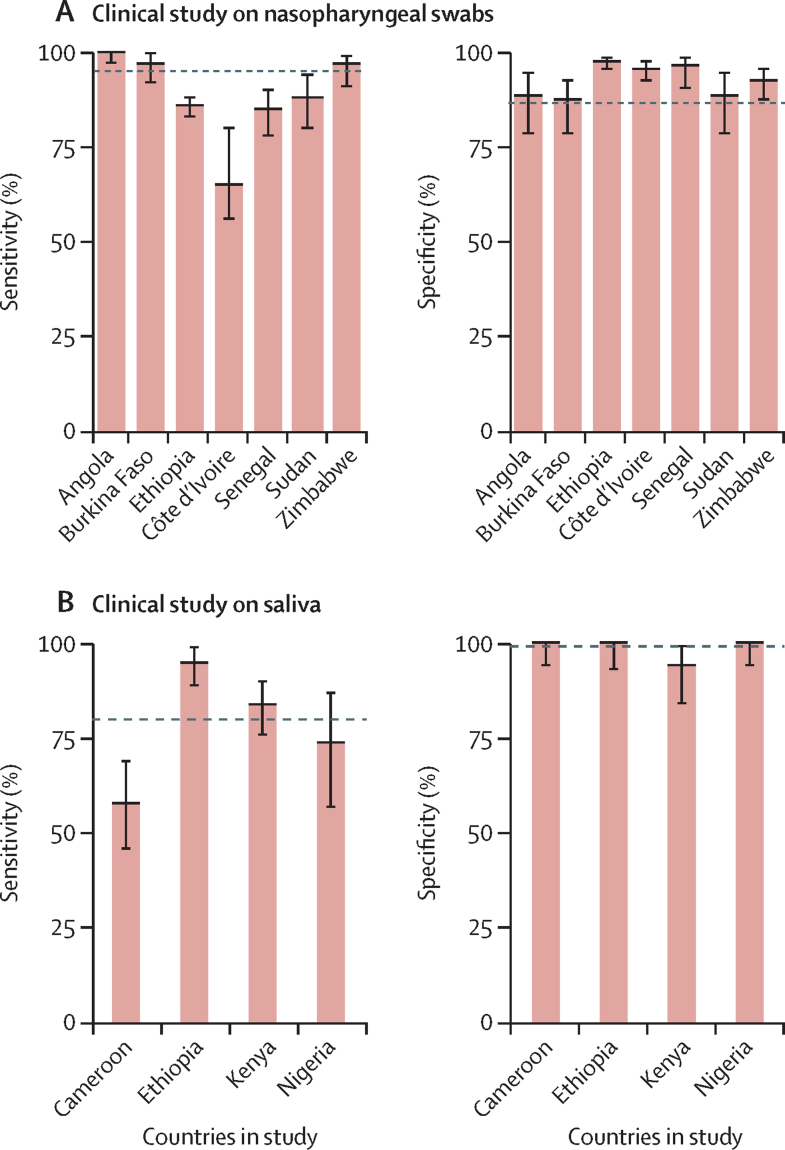
Sensitivity and specificity of colorimetric RT-LAMP with RNA extracted from nasopharyngeal swabs RT-LAMP was conducted with RNA extracted from nasopharyngeal swabs (A) or with crude saliva samples (B). Sensitivity and specificity were calculated relative to the standard of diagnosis. Dashed lines represent the overall specificity or sensitivity of RT-LAMP relative to the standard of diagnosis. Error bars indicate 95% CIs. RT-LAMP=reverse transcription loop-mediated isothermal amplification.

On the basis of these overall sensitivity and specificity results, we also calculated the positive predictive value ([PPV] ie, the probability that an individual with a positive test result has the disease) and negative predictive value ([NPV] ie, the probability that an individual with a negative test result does not have the disease) of RT-LAMP on RNA extracted from nasopharyngeal swabs, using the lowest and highest SARS-CoV-2 prevalence rates reported in Africa during the COVID-19 pandemic (appendix p 1). At a SARS-CoV-2 prevalence of 3·7% (ie, in Cameroon and Angola), the PPV was 41% (95% CI 32–46), and the NPV was 100% (100–100). In contrast, at a prevalence of 18·9% (ie, in Burkina Faso), the PPV was 81% (74–84), and the NPV was 97% (97–98). On stratification of samples on the basis of Ct values obtained by RT-qPCR, the sensitivity of RT-LAMP was lower when viral load was lower (ie, higher Ct values; appendix p 6).

In the clinical study on saliva, of 577 nasopharyngeal swabs that had corresponding saliva samples, 340 (58·9%) tested positive for SARS-CoV-2 by RT-qPCR. A higher number of inconclusive results was observed with crude saliva analysed with RT-LAMP (31 [5·4%] samples; appendix p 5) than with RNA extracted from nasopharyngeal swabs analysed with RT-qPCR (80 [2·9%] samples; appendix p 6), as variable saliva pH was noted to influence the initial colour of the RT-LAMP reaction mix as well as the colorimetric change on template amplification.

In crude saliva samples, the overall sensitivity of RT-LAMP was 80% (95% CI 75–84), specificity was 99% (96–100), and accuracy was 88% (85–90) compared with the standard of diagnosis (figure 4B, appendix p 7). Lower sensitivity for RT-LAMP conducted in Cameroon and Nigeria was attributed to the higher percentages of negative samples collected in these two countries than in the other countries and low viral loads in most of the positive cases (appendix p 7). As expected, stratification of samples on the basis of Ct assessed by RT-qPCR from swabs clearly showed that the sensitivity of the RT-LAMP to detect SARS-CoV-2 in saliva generally decreased with a decrease in viral load (appendix p 7).

Using the overall sensitivity and specificity assessed in the clinical study on saliva (appendix p 7), we calculated the PPV and the NPV using the lowest and highest SARS-CoV-2 prevalence rates reported in Africa during the COVID-19 pandemic (appendix p 1). At a prevalence of 3·7%, the PPV was 76% (95% CI 42–100), and the NPV was 99% (99–99). In contrast, with a SARS-CoV-2 prevalence of 18·9%, the PPV was 95% (82–100), and the NPV was 96% (94–96).

## Discussion

Following our previous pilot study of diagnostic accuracy,^11^ we extended testing of RT-LAMP technology to six additional African countries and seven regional laboratories across Ethiopia. This expanded assessment confirmed the high sensitivity and specificity (89% and 95%, respectively) of RT-LAMP for detection of SARS-CoV-2 with the original protocol (ie, RNA extraction from nasopharyngeal swabs followed by RT-LAMP). It should be noted that all RT-LAMP results in the previous study were benchmarked against the same RT-qPCR reference test, which was provided to participating laboratories together with the RT-LAMP kits. However, in the present study, the performance of RT-LAMP was compared with that of the standard of diagnosis in each participating centre, meaning that different RT-qPCR kits were used (with different viral targets and result interpretations). Thus, RT-qPCR results were interpreted according to the manufacturer's instructions or in agreement with nationally accepted positivity thresholds, which helps to explain the variation in sensitivity levels between countries. In addition, although we aimed to collect 300 samples per country, including 100 negative samples and 200 positive samples (of which 30% should have had a Ct >30), this target was not always possible to achieve, due to the low availability of both positive samples and samples with a high viral load.

In a similar study, 100% sensitivity and 96% specificity for detecting SARS-CoV-2 were reported for an RT-LAMP assay on samples with viral loads higher than 100 copies per reaction,^15^ which is consistent with our findings. Another study^32^ recently reported 90% sensitivity for detecting SARS-CoV-2 for a RT-LAMP assay tested on 350 RNA samples extracted from swabs, increasing to 96% for samples with Ct values lower than 30. In a systematic review^33^ comparing the analytical performances of different RT-LAMP assays for detecting SARS-CoV-2 (with or without RNA extraction), the overall sensitivity and specificity were 79% and 97%, respectively. The sensitivity of tests with extracted RNA reported in the systematic review appeared to be higher (88%) than the sensitivity of tests without RNA extraction (50%), although specificity remained high. In this study, the PPV and NPV values calculated for RT-LAMP were substantially influenced by infection prevalence, as expected, with RT-LAMP yielding a higher proportion of false positives at the lowest observed SARS-CoV-2 prevalence than it did at high prevalence, even when the specificity of the test was acceptable. At the highest SARS-CoV-2 prevalence, PPV values were higher with crude saliva than with nasopharyngeal swabs (95% *vs* 81%).

Saliva has grown increasingly popular as an alternative respiratory sample to nasopharyngeal swabs for detecting SARS-CoV-2.^8^, ^18^, ^34^ The overall sensitivity (80%) and specificity (99%) of our protocol, which includes a lysis buffer easily prepared in-house, showed an acceptable performance for RT-LAMP. Similarly, a previous study^15^ evaluated a SARS-CoV-2 RT-LAMP test directly on saliva and showed the sensitivity of the assay to be lower (85%) when crude samples were used than when extracted RNA with >100 copies per reaction was used (100% sensitivity). A slight decline in sensitivity has been reported for different RT-LAMP assays on crude extracts when RNA extraction is omitted.^22^, ^33^ Nonetheless, Huang and colleagues^35^ showed that a multiplexed RT-LAMP assay could detect as few as 1·5 copies per μL of SARS-CoV-2 in saliva, similar in sensitivity and specificity to standard RT-qPCR.

As observed previously for nasopharyngeal swabs, the variation in sensitivity of RT-LAMP with crude saliva could be attributed to low positivity rates at the time of collection in some countries, as well as high Ct values (ie, low viral loads). In addition, variations in sensitivity could also be attributed to subjective result interpretation. Colorimetric read-out, although considered a clear advantage for assay deployment in community settings, might lead to interpretation bias. Purified RNA samples might not cause this problem, as the neutral pH of elution buffer does not influence the initial colour of the reaction mix (pink), but crude saliva samples can vary in their pH, affecting the initial colour of the reaction mix and making result interpretation more challenging, especially for non-experienced personnel. This problem occurred even though the sample collection protocol instructed participants to avoid eating, drinking, or smoking for 1 h before saliva collection and included a step for rinsing the mouth beforehand. The saliva lysis buffer has a limited buffering capacity to avoid obscuring pH changes occurring during template amplification, which are the basis for the colorimetric change. Indeed, 5·4% of saliva samples gave inconclusive results. Recording the initial colour change before launching the RT-LAMP helps to reduce the risk of result misinterpretation. However, even if results were inconclusive, we strongly advised against confirming results by gel electrophoresis, as opening the tubes at the end of the RT-LAMP reaction can rapidly cause widespread contamination.

In this study, results of RT-LAMP on crude saliva samples were always benchmarked against RT-qPCR run on RNA extracted from nasopharyngeal swabs, meaning that we not only compared two different diagnostic techniques (RT-LAMP without RNA purification *vs* RT-qPCR on extracted RNA) but also two different sample types. Variable concentrations of virus across different samples should be considered when assessing the performance of diagnostic tools. In our paired samples, viral loads were generally lower in saliva than in nasopharyngeal swabs. Variability in viral loads in different matrices means that SARS-CoV-2 virus might be detected in one sample type but not the other (or vice versa), even when the same detection method is used. There have been conflicting reports regarding SARS-CoV-2 concentrations in saliva,^19^, ^36^ including a meta-analysis^37^ that found no significant differences in SARS-CoV-2 loads in saliva and oropharyngeal swabs, nasopharyngeal swabs, or sputum. Variation between studies might be attributable to factors such as differences in saliva collection methods, dilution of saliva samples after collection, stage of infection,^38^ type of SARS-CoV-2 variant,^21^, ^39^ and the time of the day when samples were collected.^40^ The theoretical and clinical limits of detection of our simplified protocol for crude saliva samples were similar to the limits for purified RNA. Unlike purified RNA, however, crude saliva samples might contain sample-derived inhibitory substances that block polymerase activity, inhibit template amplification, and result in false negatives.^41^, ^42^ To ensure sample compatibility, especially when crude saliva samples are tested, and to control for proper sample acquisition and storage, an internal control reaction should be assembled in parallel for every saliva sample.

Beyond COVID-19 testing, RT-LAMP could be suitable for pathogen detection across various settings, from well-equipped centralised laboratories to resource-limited settings and peripheral facilities at the community level.^43^ Commercial LAMP kits for detecting infectious diseases such as malaria, tuberculosis, leishmaniasis, H5N1 influenza, and dengue have been used in Zanzibar, Colombia, and Malaysia,^44^, ^45^, ^46^, ^47^ and many research groups have developed and evaluated their own in-house LAMP assays.^48^, ^49^, ^50^, ^51^, ^52^, ^53^ Encouraged by our findings, we are currently exploring the potential of RT-LAMP for detecting arboviral infections (unpublished data). Furthermore, the Africa CDC has expressed interest in evaluating LAMP assays recently developed for detecting mpox.^54^, ^55^

RT-LAMP still has some limitations. For example, it is a semi-quantitative molecular tool that, unlike the gold-standard RT-qPCR used by reference diagnostic labs, cannot reliably distinguish between high and low viral loads. Nonetheless, we believe that its diagnostic performance, together with its ease of use, short turnaround time, and lower overall price compared with RT-qPCR make it suitable to be deployed at the point of care and in low-resource settings.

### Contributors

### Data sharing

Data used in this study are available from the corresponding authors on reasonable request.

## Declaration of interests

We declare no competing interests.
