# Supplementary material for: Colorimetric RT-LAMP for SARS-CoV-2 detection from nasopharyngeal swabs or crude saliva: a multicountry diagnostic accuracy study in Africa
Source: Lancet Glob Health. 2025 Jun 25;13(7):e1258–67. doi: 10.1016/S2214-109X(25)00150-0 (PMC12208784; doi:10.1016/S2214-109X(25)00150-0)
Supplement: Supplementary appendix [file mmc1.pdf]

THE LANCET
Global Health

Supplementary appendix

This appendix formed part of the original submission and has been peer reviewed. We post it as supplied by the authors.

Supplement to: Šušnjar U, Bitew M, Ayele S, et al. Colorimetric RT-LAMP for SARS-CoV-2 detection from nasopharyngeal swabs or crude saliva: a multicountry diagnostic accuracy study in Africa. *Lancet Glob Health* 2025; **13:** e1258–67.

Appendix

Contents

Supplementary Tables 1
Supplementary Figures 8
Supplementary references 9
EXPANDIA working group 10

Supplementary Tables

Supplementary Table 1. **SARS-CoV-2 prevalence in partner countries and as reported by Africa CDC (last update on January 1st 2023) and in different publications (limited to RT-qPCR testing)**

	Africa CDC dashboard			Selected publications				
**Country**	**Total tests**	**Total cases**	**% positive tests**	**Total testes**	**Total cases**	**% positive tests**	**Period**	**Reference**
Continent	125,168,543	12,216,748	9.8					
Angola	2,875,973	104,973	3.7					
Burkina Faso	324,773	22,006	6.8	1,506	216	14.3	Sep 2021 - Feb 2022	(1)
				1,758	332	18.9	Dec 2021 - Jan 2022	(2)
Cameroon	3,363,346	124,188	3.7	671	78	11.6	Jan 2021 - April 2021	(3)
Ivory Coast	1,663,177	87,941	5.3	4,071	719	17.7	May 2020 - May 2021	(4)
Ethiopia	5,420,453	498,348	9.2		35,376	8.8	up to Apr 2022	(5)
Kenya	3,958,283	342,511	8.7	99,649	7,737	7.8	Mar 2020 - Jun 2021	(6)
Nigeria	5,708,974	266,381	4.7		161,737	9.6	Mar 2020 - Mar 2021	(7)
Senegal	1,294,129	88,900	6.9	8,207	970	11.8	Jul 2020 - Dec 2021	(8)
				4,146	188	4.5	Mar 2021 - May 2021	(9)
Zimbabwe	2,578,048	259,628	10.1	92,299	8,099	8.8	Mar 2020 - Oct 2020	(10)
Slovenia				4,697,483	326,155	6.9	Sept 2021 Oct 2021 Jan 2022	unpublished

Supplementary Table 2. **Cohort characteristics (Ljubljana, Slovenia)**

**Cohort characteristics**	**Count (percentage in %)**
Female	203/338 (60.1)
Median age (years)	35
Vaccinated	126/275* (45.8)
1. dose	24/275 (8.7)
2. dose	93/275 (33.8)
3. dose	9/275 (3.3)
Symptoms	273/283* (96.5)
Median disease duration (days)	3 (1-14)

* Total number of participants that provided an answer

Supplementary Table 3. **Methods employed by each participating laboratory testing RT-LAMP assay on RNA extracted from swabs**

Country	Site of collection	Period of collection	Laboratory	Swab type	RNA extraction method	RT-qPCR method	Positivity interpretation	Ethical approval number (Regulatory body)
Angola	Community settings (markets, residences); hospital settings (health medical centres and Hospital Américo Boavida); research institute (National Institute for Health Research (INIS))	August 2020 - August 2022	Faculdade de Medicina da Universidade Agostinho Neto (FMUAN)	Nasopharyngeal swabs	Automatically using KingFisher Duo Prime machine (ThermoFisher) and MagMax Viral/Pathogen Isolation Kit (ThermoFisher) or manually using Nucleic Acid Extraction Rapid Kit (Bioperfectus Technologies)	7500 Fast Dx Real-Time PCR System (ThermoFisher). Manually with the kit including TapPath Covid-19 CE-IVD RT-QPCR (ThermoFisher) and COVID-19 Coronavirus Real Time PCR Kit (Bioperfectus Technologies)	Samples in which a positive signal was detected at least in two of three gene targets (ORF1ab, S and N gene) were considered positive irrespective of the Ct value	Deliberação Nº 19/2021 (Instituto regulador - Faculdade de Medicina da Universidade Agostinho Neto)
Burkina Faso	Ouagadougou and Bagassi region	August 2021 and September 2022	National Flu Reference Laboratory (LNR-G) at the Institute for Research in Health Sciences (IRSS)	Nasopharyngeal and oropharyngeal swabs	Manually using QIAmp Viral RNA Mini Kit (Qiagen)	Manually using TaqPath™ COVID19 CEIVD RTPCR Kit (Applied Biosystems) or STANDARD M nCoV Real-Time Detection kit (SD Biosensor)	One of two targets (ORF1ab and N gene) should give a positive signal. Ct of 36 was considered positivity threshold	2022-08-178 (Ethics Committee for Health Research; Ministry of Health, Public Hygiene - Ministry of Higher Education, Research and Innovation)
Ivory Coast	Pasteur Institute of Côte d'Ivoire and Health district of Cocody, Abidjan	April 2022 - October 2022	Molecular biology Lab Platform, Pasteur Institute of Côte d'Ivoire	Nasopharyngeal swabs	Manually using QIAmp Viral RNA mini kit (Qiagen)	QuantStudio ™ 5 thermocycler (Applied Biosystems) using STANDARD M nCoV Real-Time Detection kit (SD Biosensor)	Both gene targets (E and ORF1ab gene) show a positive signal regardless the Ct value obtained	CNSEVSRef-28-22 (Comite national d'Ethique des sciences de la vie et de la santé)
Ethiopia	Collected in laboratory conducting the study; samples analysed by BETin were collected by Ethiopian Public Health Institute (EPHI)	November 2021 - September 2022	Armour Hansen Research Institute (AHRI), Ethiopian Public Health Institute (EPHI), Saint Paul Hospital and Millennium Medical College (SPHMMC), BETin, Hawassa University (HU), Animal Health Institute (AHI) and Amhara Public health Institute (APHI)	Nasopharyngeal swabs	Manually using QIAmp Viral RNA mini kit (Qiagen)	Different PCR kits	The Ct of 39 (ORF1ab gene) was considered a positivity threshold	EBTI/002/2022 (Ethiopian Food Drug authority)
Senegal	Health districts of the Thies region	January 2021 - October 2022	The Institut de Recherche en Santé de Surveillance Epidemiologique et de Formation (IRESSEF)	Nasopharyngeal swabs	Automatically using KingFisher with 96 PCR head machine (ThermoFisher) and Magmax RNA extraction kit (Applied Biosystems)	Automatically using CFX-96 (Bio-Rad) or QuantStudio 5 thermocyclers (Applied Biosystem) using 3 different kits; Allplex SARS-CoV-2 Assay (Seegene), Nucleic Acid Diagnostic Kit (2019-nCov) (Sansure Biotech) and TaqPath™ 1-Step RT-qPCR Master (Applied Biosystems)	The Ct of 39 was used as a positivity threshold	SEN20/30 (Comité National d'Ethique pour la Recherche en Santé)
Sudan	National laboratory for Public Health and Alfaisal Private Laboratory, Khartoum	January 2021 - December 2022	Tropical Medicine Research Institute in Khartoum	Nasopharyngeal swabs	Manually using ZYMO Viral RNA Extraction kits (Zymo Research)	Automatically using CFX 96 thermocycler (Biorad) or Geneamp 9700 PCR (Applied Biosystems) using Allplex qRT-QPCR for SARS CoV-2 detection kit (Seegene)	The Ct of 40 was used as a positivity threshold. At least one gene target needed to be detected (S gene, RdRp gene or N-gene)	TMRI/EA/133.22 (Tropical Medicine Research Institute\ National Centre for Research), stored samples
Zimbabwe	National Microbiology Reference Laboratory (NMRL) biobank	April 2022 - December 2022	National Microbiology Reference Laboratory (NMRL) in Harare	Nasopharyngeal swabs	Automatically using EasyMag machine and Nuclisens EasyMag Total nucleic acid extraction kit (Biomerieux)	Manually using BGI SARS-COV-2 amplification and detection kit and the Gentier96E real-time PCR machine	The Ct of 39 was used as a positivity threshold (ORF1ab)	SMCHE181023/89 (Sally Mugabe Central Ethics Review Committee)

Supplementary Table 4. **Methods employed by each participating laboratory testing RT-LAMP assay on crude saliva samples in parallel to RNA extracted from paired swab samples**

Country	Site of collection	Period of collection	Laboratory	RNA extraction	RT-qPCR	Positivity interpretation	Ethical approval (Regulatory body)
**Cameroon**	Chantal Biya International Reference Center (CIRCB), Biyem assi, Efoulan, Yaoundé Hospitals	May 2022 - December 2022	the virology laboratory of CIRCB	Manually by RNA/DNA purification kit (DaAn Gene)	Manually using QuantStudio 5 thermocycler (Applied Biosystems) with the Detection kit for 2019 novel coronavirus (2019-ncoV) RNA (PCR-Fluorescence probing) (DaAn Gene)	The Ct of 37(the average of all gene targets) was used as a positivity threshold (national positivity threshold)	2022/01/1430/CE/CNERSH/SP (National Ethics Committee for Research on Human Health)
**Ethiopia**	different hospitals by the Ethiopian Public health Institute (EPHI)	June 2022 – August 2022	Bio and Emerging Technology Institute (BETin)	Manually using QIAmp Viral RNA mini kit (Qiagen)	Manually by Beijing Genomic Institute Real-Time Fluorescent RT-qPCR kit for detecting 2019-nCoV (SARS-CoV-2) (BGI)	The Ct of 38 was used as a positivity threshold (ORF1ab gene)	EBTI/002/2022 (Ethiopian Food Drug authority)
**Kenya**	the central laboratory, Nairobi	February 2022 - September 2022	the Centre for Virus Research and Centre for Biotechnology Research, Kenya Medical Research Institute (KEMRI)	Manually using QIAmp Viral RNA mini kit (Qiagen)	anually using The Liferiver Novel Coronavirus (2019-nCoV) Real Time Multiplex RT-qPCR Kit (Shanghai ZJ Bio-Tech)	The Ct of 41 was used as a positivity threshold (at least two targets – ORF1ab, N, E, needed to have a positive signal)	KEMRI/SERU/CBRD/218/4131 (KEMRI Scientific and Ethical Review Unit)
**Nigeria**	University of Maiduguri Teaching Hospital	January 2022 - February 2023	the University of Maiduguri Teaching Hospital Molecular Laboratory (A covid 19 laboratory optimized by Nigeria Center for Disease Control (NCDC)	Manually using QIAmp Viral RNA mini kit (Qiagen)	mManually using the Liferiver Novel Coronavirus (2019-nCoV) Real Time Multiplex RT-qPCR Kit (Shanghai ZJ Bio-Tech) and TaqPath™ COVID19 CEIVD RTPCR Kit (Applied Biosystems)	One of two gene targets (ORF1ab and N gene) needed to give a positive signal	UMTH/REC/1117 (Medical Laboratory Science Council of Nigeria)

Supplementary Table 5. **Swab samples collected per country.** Sample number, percentage of positive samples (as assessed by RT-qPCR) and inconclusive LAMP results

Country	Samples	Positive samples	% of positive samples	LAMP inconclusives	% of inconlusives
Angola	300	208	69.3	23	7.7
Burkina Faso	219	122	55.7	0	0.0
Ivory Coast	397	34	8.6	0	0.0
Senegal	253	156	61.7	0	0.0
Sudan	198	122	61.6	33	16.7
Zimbabwe	249	96	38.6	0	0.0
Ethiopia	1158	784	67.7	24	2.1
**Overall**	**2774**	**1522**	**54.9**	**80**	**2.9**

Supplementary Table 6. **Performance of RT-LAMP in the detection of SARS-CoV-2 RNA from NP swabs as assessed by each participating country.** Diagnostic parameters, namely: sensitivity, specificity, accuracy of the index assay, compared with the reference, were derived from the confusion matrix as previously described. The Cohen's Kappa coefficient was used to measure inter-rater reliability. p-values were calculated to test statistical significance of the test. Intervals with 95% confidence (95% CI) are also shown. N is the number of samples that were considered for the analysis that gave conclusive results.

Country	N	Sensitivity % (95% CI)	Specificity % (95% CI)	Accuracy % (95% CI)	Cohen's kappa (95% CI)
Angola	277	99 (97-100)	87 (79-94)	96 (94-98)	91 (86-97)
Burkina Faso	219	97 (92-99)	88 (79-93)	93 (88-96)	85 (78-92)
Ivory Coast	397	65 (46-80)	96 (93-98)	93 (90-95)	58 (43-73)
Senegal	253	85 (78-90)	97 (91-99)	89 (85-93)	78 (70-86)
Sudan	165	88 (80-94)	89 (79-95)	89 (83-93)	77 (67-87)
Zimbabwe	249	97 (91-99)	93 (88-96)	94 (90-96)	88 (82-94)
Ethiopia	1134	86 (83-88)	98 (96-99)	90 (88-93)	78 (74-82)
**Overall**	**2694**	**89 (87-90)**	**95 (93-96)**	**92 (90-93)**	**83 (81-85)**

Supplementary Table 7. **Sensitivity values determined for samples stratified by viral concentration (Ct values as assessed by RT-qPCR on RNA extracted from swabs)**

Stratification	Angola		Burkina Faso		Ivory cost		Senegal		Sudan		Zimbabwe		Ethiopia	
	N	Sensitivity % (95% CI)	N	Sensitivity % (95% CI)	N	Sensitivity % (95%CI)	N	Sensitivity % (95% CI)	N	Sensitivity % (95% CI)	N	Sensitivity % (95% CI)	N	Sensitivity % (95% CI)
CT<25	129	100 (97-100)	75	97 (90-100)	15	93 (68-100)	58	97 (88-100)	7	100 (59-100)	40	100 (91-100)	365	93 (90-96)
CT<30	201	100 (98-100)	101	98 (93-100)	21	90 (70-99)	108	97 (92-99)	28	89 (72-98)	69	100 (95-100)	551	92 (89-94)
CT≥30	5	83 (36-100)	21	90 (70-99)	13	23 (5-54)	48	56 (41-71)	65	88 (77-95)	27	89 (71-98)	209	69 (62-75)
CT<35	206	99 (97-100)	122	97 (92-99)	31	68 (49-83)	138	94 (89-97)	68	85 (75-93)	89	100 (96-100)	693	88 (86-91)
25≤CT<30	73	100 (95-100)	30	100 (88-100)	6	83 (36-100)	50	98 (89-100)	21	85 (62-97)	29	100 (88-100)	186	88 (83-93)
30≤CT<35	4	75 (19-99)	17	88 (64-99)	10	20 (3-56)	30	83 (65-94)	40	82 (67-93)	20	100 (83-100)	142	76 (68-83)
35≤CT<40	0	na	0	na	3	33 (1-91)	18	11 (1-35)	25	96 (79-100)	7	57 (18-90)	67	54 (41-67)

Supplementary Table 8. **RT-qPCR of saliva samples following RNA extraction per country**

Country	Samples	Positive samples	% of positive samples	LAMP inconclusive	% of inconclusive
Cameroon	150	77	51.3	9	6.0
Ethiopia	150	94	62.7	9	6.0
Kenya	176	125	71.0	5	2.8
Nigeria	101	44	43.6	8	7.9
**Overall**	**577**	**340**	**58.9**	**31**	**5.4**

Supplementary Table 9. **Diagnostic performance of RT-LAMP to detect SARS-CoV-2 on crude saliva samples by each participating country.** Diagnostic parameters namely: sensitivity, specificity, accuracy of the index assay (RT-LAMP) compared with the reference (RT-qPCR), were derived from the confusion matrix as previously described. The Cohen's Kappa coefficient was used to measure inter-rater reliability. P-values were calculated to test statistical significance of each test. Intervals with 95% confidence (95% CI) are also shown. N is the number of samples giving conclusive results that were considered for the analysis

Country	N	Sensitivity % (95% CI)	Specificity % (95% CI)	Accuracy % (95% CI)	Cohen's kappa (95% CI)
Cameroon	141	58 (46-69)	100 (94-100)	77 (70-84)	55 (42-69)
Ethiopia	141	95 (89-99)	100 (93-100)	97 (93-99)	94 (88-100)
Kenya	171	84 (76-90)	94 (84-99)	87 (81-92)	72 (61-83)
Nigeria	93	74 (57-87)	100 (94-100)	89 (81-95)	77 (63-90)
**Overall**	**546**	**80 (75-84)**	**99 (96-100)**	**88 (85-90)**	**75 (70-81)**

Supplementary Table 10. **Sensitivity of RT-LAMP on saliva according to viral concentration Ct values as assessed by RT-qPCR on RNA extracted from swabs**

Stratification	Cameroon		Ethiopia		Kenya		Nigeria	
	N	Sensitivity % (95% CI)	N	Sensitivity % (95% CI)	N	Sensitivity % (95%CI)	N	Sensitivity % (95% CI)
CT<25	32	94 (79-99)	59	100 (94-100)	60	88 (77-95)	5	80 (28-99)
CT<30	48	83 (70-93)	74	100 (95-100)	89	84 (75-91)	22	77 (55-92)
CT≥30	28	14 (4-33)	14	71 (42-92)	31	84 (66-95)	16	69 (41-89)
CT<35	67	66 (53-77)	81	100 (96-100)	113	85 (77-91)	36	72 (55-86)
25≤CT<30	16	62 (35-85)	15	100 (78-100)	29	76 (56-90)	17	76 (50-93)
30≤CT<35	19	21 (6-46)	7	100 (59-100)	24	88 (68-97)	14	64 (35-87)
35≤CT<40	9	0	7	43(10-82)	7	71 (29-96)	2	100 (16-100)

Supplementary Figures

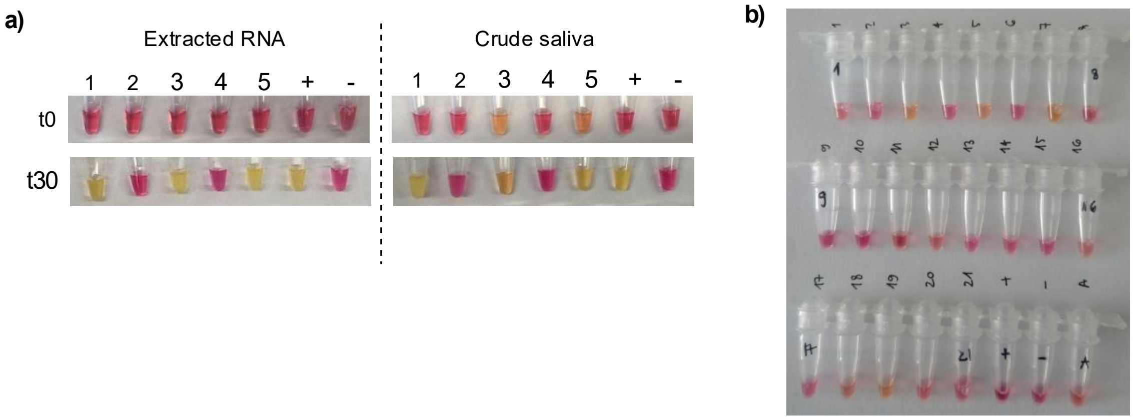

Supplementary Figure 1. **Exemplary RT-LAMP experiment.**

a) An example of an RT-LAMP experiment conducted using 5 samples; either extracted RNA (left) or lysed saliva (right), along with corresponding controls. These controls include synthetic N gene (positive control) and nuclease-free water (negative control). The color was recorded immediately after sample addition (t0) and following a 30-minute incubation at 65°C (t30). Samples 3 and 5 (right panel) show inconclusive results. When crude saliva yields inconclusive results, RNA purification should be performed, as saliva pH influences color formation.

b) The color of the pH-sensitive mastermix may shift from pink to orange when lysed saliva samples with varying pH levels are added (t=0).

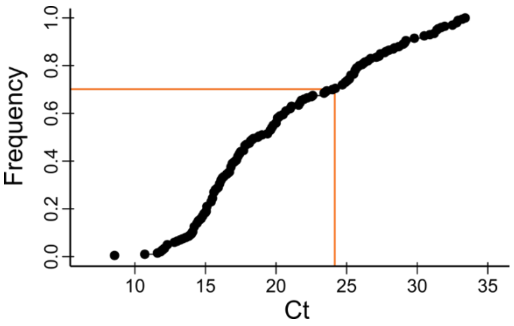

Supplementary Figure 2. **Distribution of Ct values among clinical samples.**

The cumulative frequency plot shows Ct values among SARS-CoV-2 positives as diagnosed by RT-qPCR (CE IVD LightMix kit SARS-CoV-2 E+N UBC) performed on RNA samples extracted from NP swabs.

Supplementary references

1. Cissé A, Lingani M, Tarnagda Z, Tao M, Nana S. Prevalence of COVID-19 at the Wahgnion-Gold mining site in Burkina Faso and use of RT-PCR initial cycle threshold to monitor the dynamics of SARS-CoV-2 load." African Journal of Clinical and Experimental Microbiology 24.1 (2023): 24-31. Clinical and Experimental Microbiology. 2023;1(24):24–31.

2. Soubeiga ST, Institut de Recherche en Sciences de la Santé (IRSS) /Laboratoire de Recherche Biomédicale (LaReBio), Ouagadougou, Burkina Faso, Charlotte K, University Joseph Ki-Zerbo, Laboratory of Molecular Biology and Genetics (LABIOGENE), Ouagadougou, Burkina Faso, Zoure AA, Institut de Recherche en Sciences de la Santé (IRSS) /Laboratoire de Recherche Biomédicale (LaReBio), Ouagadougou, Burkina Faso, et al. SARS-CoV-2 Variants Screening in Burkina Faso. JoMMID. 2022 Sep 1;10(3):135–40.

3. Nguwoh PS, Ngounouh CT, Essomba RG, Ngo Likeng JL, Akenji Mboringong B, Halilou I, et al. Prevalence of SARS-CoV-2 and Associated Factors Among Individuals During the Second Wave of Infection in Yaounde, Cameroon. EJMED. 2024 Sep 25;6(5):35–40.

4. Anoh EA, Wayoro O, Monemo P, Belarbi E, Sachse A, Wilkinson E, et al. Subregional origins of emerging SARS-CoV-2 variants during the second pandemic wave in Côte d'Ivoire. Virus Genes. 2023 Jun;59(3):370–6.

5. Gedefie A, Tilahun M, Fiseha M, Alemayehu E, Shibabaw A, Bisetegn H, et al. Epidemiology of SARS-CoV-2 Infection in Ethiopia: A Systematic Review and Meta-Analysis. COVID. 2023 May 4;3(5):703–14.

6. Nyagwange J, Ndwiga L, Muteru K, Wamae K, Tuju J, Testing Team C, et al. Epidemiology of COVID-19 infections on routine polymerase chain reaction (PCR) and serology testing in Coastal Kenya. Wellcome Open Res. 2022 Feb 23;7:69.

7. Oleribe O, Olawepo O, Ezechi O, Osita-Oleribe P, Fertleman M, Taylor-Robinson SD. Describing the Epidemiology of COVID-19 in Nigeria: An Analysis of the First Year of the Pandemic. Journal of Health Care for the Poor and Underserved. 2022;33(1):33–46.

8. Ba AA, Coppée R, Dieng A, Manneh J, Fall M, Gueye K, et al. Genomic epidemiology of SARS-CoV-2 in Senegal in 2020-2021. J Infect Dev Ctries. 2024 Jun 30;18(06):851–61.

9. Moustapha M, Ibrahima D, Mamadou D, Marouba C, Moctar G, Aminata M, et al. Evaluation of the LumiraDx SARS-CoV-2 antigen assay for large-scale population testing in Senegal. Int J Clin Virol. 2022 Jan 5;6(1):001–6.

10. Mashe T, Takawira FT, De Oliveira Martins L, Gudza-Mugabe M, Chirenda J, Munyanyi M, et al. Genomic epidemiology and the role of international and regional travel in the SARS-CoV-2 epidemic in Zimbabwe: a retrospective study of routinely collected surveillance data. The Lancet Global Health. 2021 Dec;9(12):e1658–66.

EXPANDIA working group

Name	Surname
Nasser Caiombo	Calumbuana
Elka	Kay
Valdemar José	Mateus
Joana Paulo	Paixão
Faustino Manuel Savoloto	Ramos
Joaquim Carlos Vicente	Van-Dunem
Maria Madalena	Chimpolo
Assana	Cisse
W. O. Benjamin	Kabore
Moussa	Lingani
Evelise Augusto	Machado
Jean Bienvenue	Ouoba
Grissoum	Tarnagda
Zékiba	Tarnagda
Collins	Ambe Chenwi
Aude Christelle	Ka'e
Aurelie Minelle	Ngueko Kengni
Aissatou	Abba
Grace	Beloumou
Takou	Desire
Sandrine	Djupsa
Naomi-Karell	Etame
Nadine Nguendjoung	Fainguem
Ezechiel Ngoufack	Jagni Semengue
Nnomo Zam	Marie Kryste
Larissa Gaëlle	Moko Fotso
Evariste	Molimbou
Alexis	Ndjolo
Alex Durand	Nka
Michel Carlos	Tchouaket Tommo
Joseph	Fokam
Adugna	Abera
Getachew	Abichu Duressa
Kominist Asmamaw	Anley
Gadissa	Bedada Hundie
Hailu	Dadi
Feleke	Mekonnen
Yared	Merid
Andaragachew	Mulu
Kassahun	Tesfaye
Keyru	Tuki
Gizachew	Yismaw
Samuele	Ayele

Molalegne	Bitew
Jean Luc Frejus	Aboh Kamenan
Serges	Camara
M. Anicet	Ebou
Sylvie Mireille	Kouamé-Sina
Dosso	Mireille
Aboubacar	Sylla
Albert Konan	Yavo
Solange	Kakou-Ngazoa
Wallace Dimbuson	Bulimo
Janet Masitsa	Majanja
Samwel Lifumo	Symekher
Eric Lelo	Agola
Ibrahim	Kidda
Tijani	Abdulkadir
Ahmed	Ahidjo
Musa Joseph	Bamaiyi
Bamidele Soji	Oderinde
Marycelin Mandu	Baba
Mame Matar	Diop
Ndeye Dieynaba	Diouf
Nafissatou	Leye
Djirbil	Wade
Asrar	Elegail
Rasheeda	H. A. Ahmed
Hanan	Mohamed
Musab	Elnegoumi
Nuha	Yousif
Shahinaz	Bedri
Mohamed-Ahmed	Elnour
Fathi	A. Mansour
Mubarak	Mustafa
Salaheldein	Elzaki
Lubna	Tagelsir Karamalla
David	Amini
Ezekiel	Dhitima
Hlanai	Gumbo
Agnes	Juru-Chibango
Tapfumanei	Mashe
Charles	Nyagupe
Raiva	Simbi
Lucia	Sisya
Kenneth	Mudzai Maeka
Tea	Carletti
Laura	De Conti
Erica	Bussani
Mariana	Ulinici

Urša	Šušnjar
Alessandro	Marcello
